# SUMO and SUMOylation Pathway at the Forefront of Host Immune Response

**DOI:** 10.3389/fcell.2021.681057

**Published:** 2021-07-14

**Authors:** Sajeev T. K., Garima Joshi, Pooja Arya, Vibhuti Mahajan, Akanksha Chaturvedi, Ram Kumar Mishra

**Affiliations:** ^1^Nups and SUMO Biology Group, Department of Biological Sciences, IISER Bhopal, Bhopal, India; ^2^National Centre for Cell Science, Savitribai Phule Pune University, Pune, India

**Keywords:** SUMOylation, Ubc9, pathogen, interferon pathway, host immune response, immune cell development

## Abstract

Pathogens pose a continuous challenge for the survival of the host species. In response to the pathogens, the host immune system mounts orchestrated defense responses initiating various mechanisms both at the cellular and molecular levels, including multiple post-translational modifications (PTMs) leading to the initiation of signaling pathways. The network of such pathways results in the recruitment of various innate immune components and cells at the site of infection and activation of the adaptive immune cells, which work in synergy to combat the pathogens. Ubiquitination is one of the most commonly used PTMs. Host cells utilize ubiquitination for both temporal and spatial regulation of immune response pathways. Over the last decade, ubiquitin family proteins, particularly small ubiquitin-related modifiers (SUMO), have been widely implicated in host immune response. SUMOs are ubiquitin-like (Ubl) proteins transiently conjugated to a wide variety of proteins through SUMOylation. SUMOs primarily exert their effect on target proteins by covalently modifying them. However, SUMO also engages in a non-covalent interaction with the SUMO-interacting motif (SIM) in target proteins. Unlike ubiquitination, SUMOylation alters localization, interactions, functions, or stability of target proteins. This review provides an overview of the interplay of SUMOylation and immune signaling and development pathways in general. Additionally, we discuss in detail the regulation exerted by covalent SUMO modifications of target proteins, and SIM mediated non-covalent interactions with several effector proteins. In addition, we provide a comprehensive review of the literature on the importance of the SUMO pathway in the development and maintenance of a robust immune system network of the host. We also summarize how pathogens modulate the host SUMO cycle to sustain infectability. Studies dealing mainly with SUMO pathway proteins in the immune system are still in infancy. We anticipate that the field will see a thorough and more directed analysis of the SUMO pathway in regulating different cells and pathways of the immune system. Our current understanding of the importance of the SUMO pathway in the immune system necessitates an urgent need to synthesize specific inhibitors, bioactive regulatory molecules, as novel therapeutic targets.

## Introduction

Multifaceted interactions between the pathogens and the host immune system are among the most dynamic interplay in nature. While the host immune system tries to remove the pathogen efficiently, pathogens exploit host machinery for their benefit to escape the immune response. Moreover, pathogens keep evolving, creating enormous and unseen challenges for the immune system (Schluger and Rom, 1998; Travis, 2009; Chaplin, 2010; Ramadan et al., 2017). For efficient removal of the pathogens, the host immune system utilizes a wide variety of cellular and molecular mechanisms at both post-transcriptional and post-translational levels. Many of the post-translational mechanisms result in signal-driven modification of the target proteins in host cells. It includes phosphorylation, ubiquitination, nitrosylation, and oxidation. Recent findings indicate that in addition to being regulated by these post-translational modifications (PTMs), host proteins involved in immune responses undergo modification by a family of ubiquitin-like proteins (Ubls). The most prominent Ubl is the small ubiquitin-related modifier (SUMO), and the modification process is called SUMOylation. SUMOylation has been shown to regulate many cellular processes, including signal transduction, stress response, autophagy, nuclear-cytosolic transport, transcriptional program, protein stability, and cell cycle regulation (Eifler et al., 1998; Raman et al., 2013; Eifler and Vertegaal, 2015; Enserink, 2015). SUMOylation is a highly dynamic and reversible process that employs an array of proteins. Some of these proteins facilitate target protein SUMOylation steps, and others assist in the SUMO maturation and deconjugation processes. The precise role of SUMO proteins and associated pathways in modulating host immunity is relatively lesser known. We are beginning to appreciate that SUMOylation of immune response modulating proteins leads to the alteration of their function, activity, and localization, which might influence the disease outcome.

## SUMO Proteins and the SUMO Cycle

SUMOs, a family of ∼10- to 12-kDa proteins, are present in all eukaryotes and are highly conserved from yeast to humans. Lower eukaryotes like *Saccharomyces cerevisiae* have a single SUMO protein, Smt3p, but mammals ubiquitously express three major paralogs SUMO-1, SUMO-2, and SUMO-3. Recent evidence indicates two more paralogs SUMO-4 and SUMO-5, in mammalian cells (Raman et al., 2013; Liang et al., 2016; Baczyk et al., 2017; Li et al., 2017). SUMO-2 and SUMO-3 are ∼95% identical but share only ∼45% similarity to SUMO-1. Both SUMO-2 and SUMO-3 can form conjugated chains through a single conserved acceptor lysine, resulting in polySUMOylation. An ultradeep study identifying SUMO targets under normal and proteostatic stress suggested that SUMO-1 can also form chains, and many more lysines on the SUMO-2 surface can be utilized for chain formation (Hendriks et al., 2017). Semiquantitative immunoblot analysis on COS-7 cell lysates indicated that the overall cellular concentrations of mature and conjugated SUMO-2/3 forms are greater than that of SUMO-1 (Saitoh and Hinchey, 2000; Vertegaal et al., 2006).

**FIGURE 1:**
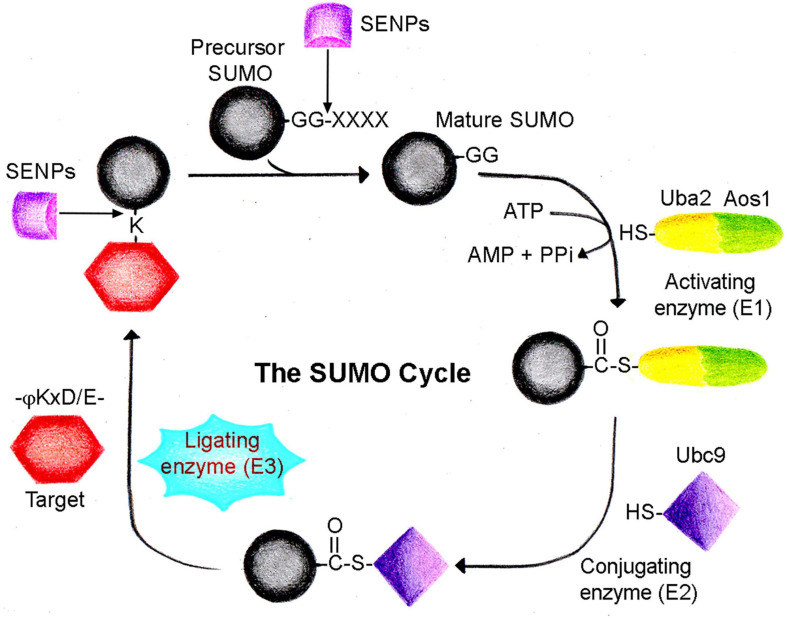
The SUMO cycle. SUMO is conjugated to a target protein following a cascade of enzyme-catalyzed reactions. Precursor SUMO is processed by sentrin proteases (SENPs) to generate the mature SUMO form. Heterodimeric SUMO activating enzyme (Uba2/Aos1, E1) utilizes ATP to form a thioester complex with mature SUMO. In the next step, SUMO is transferred to the conjugating enzyme (Ubc9, E2), forming another thioester complex. Eventually, SUMO is conjugated to the target lysine residue often present in a –ψKxD/E- motif with the dispensable use of ligating enzyme (E3). The SUMO cycle completes when conjugated SUMO is recycled again by SENP family proteases.

The SUMOylation cascade is mechanistically similar to ubiquitination. However, the SUMOylation cascade enzymes are unique and differ from the ones involved in ubiquitination (Geiss-Friedlander and Melchior, 2007; Wang and Dasso, 2009; Gareau and Lima, 2010; Pichler et al., 2017). SUMO proteins are translated as inactive precursor proteins, which are processed by a family of proteases known as ubiquitin-like-protein specific proteases (Ulps) in yeast and sentrin-specific proteases (SENPs) in mammals (Mukhopadhyay and Dasso, 2007). SUMO precursors mature through SENP-mediated processing to display the C-terminal di-glycine (-GG) motif, an inevitable step required for SUMO conjugation. Mature SUMOs get activated and form an adenylate adduct with the heterodimeric E1 enzyme Uba2/Aos1 (SAE1/2) in an ATP-dependent manner. SAE1/2 catalyzes the formation of a high-energy thioester bond between the C-terminal SUMO and active site cysteine of SAE1/2. This activated SUMO sequentially transferred to cysteine present in the active site of Ubc9 (E2 enzyme) (Bernier-Villamor et al., 2002; Capili and Lima, 2007; Knipscheer et al., 2007; Wilkinson and Henley, 2010). Activated SUMO is conjugated to a target lysine often present within a consensus sequence ψ-K-X-D/E (ψ—large hydrophobic amino acid, X—any amino acid) with the help of E2 conjugating enzyme Ubc9 (Johnson et al., 1997; Lois and Lima, 2005; Hochstrasser, 2010). SUMO conjugation is effectively an isopeptide bond formed between the carboxy terminus of SUMO and the ε-amino group of the lysine residue (Figure 1). Another class of proteins in the SUMO pathway is termed the SUMO E3 ligases, which include members of the PIAS/Siz family, RING domain and HECT domain proteins, and several proteins like RanBP2 and Pc2. SUMO E3 ligases act in concert with Ubc9 to facilitate the conjugation of SUMO to the target proteins under physiological conditions (Hochstrasser, 2001; Gareau and Lima, 2010). Apart from the ligase function, the proteins of SUMO E3 ligases may act in a SUMO E3 ligase-independent manner to regulate diverse functions, including gene expression, signal transduction, genome maintenance, and DNA repair (Pichler et al., 2002; Andrews et al., 2005; Zhao and Blobel, 2005; Geiss-Friedlander and Melchior, 2007).

In addition to covalent SUMO modification, the target protein or interactor can also engage in a non-covalent interaction with SUMOs facilitated by SUMO-interacting motifs (SIMs). SIMs, present in different proteins, can be of different types and play a central role in finding and interacting with SUMO. They generally contain a hydrophobic core with a sequence of (V/I)-X-(V/I)-(V/I) flanked by negatively charged amino acids. The prominent interaction has been shown to occur between the hydrophobic core of SIM and the surface region of SUMO, while the adjacent acidic residues have been suggested to contribute to the interaction affinity (Hecker et al., 2006; Kerscher, 2007; Gareau and Lima, 2010).

The covalent attachment of SUMO is a dynamic and reversible process. Ulps/SENPs helping in the SUMO maturation are also responsible for the deconjugation of SUMO from the target proteins. Mammals have a total of six SENPs termed SENPs 1–3 and 5–7. SENPs 1–3 and 5 are more similar to Ulp1p than Ulp2p and primarily deconjugate the SUMOylated proteins. However, SENPs 6 and 7 are more Ulp2-like and carry out poly-SUMO chain editing where SUMO monomers are sequentially removed from the polymeric SUMO chains formed on the target proteins (Li and Hochstrasser, 1999, 2000; Cheng et al., 2006; Mukhopadhyay et al., 2006; Hay, 2007; Mukhopadhyay and Dasso, 2007; Lima and Reverter, 2008; Potts, 2009).

## Role of SUMO Machinery in Immune Cell Development and Differentiation

### Immune Cell Development and Signaling

Invasion of the pathogen initiates a complex and orchestrated immune response in the host where many different types of immune cells and components get activated to remove the pathogen efficiently. Immune responses come under two broad categories, innate and adaptive. Innate immunity involves a diverse array of cells such as neutrophils, macrophages, dendritic cells, and NK cells, commonly categorized as myeloid cells, each of which plays a distinct role. Innate immune responses are non-specific. However, they are mounted rapidly after the pathogen encounter. On the other hand, adaptive immunity, which is slightly delayed, is highly specific and crucial in generating memory for the pathogen. Adaptive immunity is conferred by cells of the lymphoid lineage, B and T cells. Memory responses generated by B and T cells keep evolving, resulting in stronger and quicker responses to pathogens in subsequent encounters.

The pathogen’s presence is sensed by the pattern recognition receptors (PRRs), mainly present in various innate immune cells, such as macrophages, dendritic cells, and neutrophils. Innate immune cells express a vast array of PRRs present on the cell surface, cytoplasm, and intracellular organelles. Among the most commonly studied PRRs are Toll-like receptors (TLRs) and transmembrane proteins, which are present on the cell surface and intracellular compartments. Another class of PRR is the cytoplasmic RNA helicases, RIG-I and MDA-5, that sense the presence of cytoplasmic RNA viruses. PRRs on the innate immune cells recognize pathogens through the molecular patterns commonly referred to as pathogen-associated molecular patterns (PAMPs), which initiate a signaling cascade leading to multiple simultaneous events. It includes increased phagocytosis of the pathogen, processing, and presentation on the MHC II, and induction of proinflammatory cytokines and interferons. Collectively, these events initiate the activation of adaptive immune cells, B cells, and T cells. Antigen-specific B and T cells undergo clonal expansion and differentiation into effector cells. B cells differentiate into memory cells and antibody-secreting plasma cells. Both CD8^+^ cytotoxic T cells and CD4^+^ helper T cells differentiate into many different subtypes depending on the cytokine milieu in a context-dependent manner. The interplay between the innate and adaptive immune cells ultimately results in the efficient removal of the pathogens and the generation of the subsequent memory response (Beutler, 2004; Pascual et al., 2005; Hannoun et al., 2016; Khan et al., 2016; Adorisio et al., 2017).

While an adequate immune response is required to combat the pathogen, the inadequate one results in disease susceptibility. Moreover, the hyperactivation of the host immune response results in autoimmune and autoinflammatory disorders. These observations highlight that immune cells and pathways need to be tightly regulated at transcriptional and translational levels. Cells of myeloid and lymphoid lineages originate from the pluripotent hematopoietic stem cells (HSCs). HSCs differentiate into common myeloid progenitor (CMP) and common lymphoid progenitor (CLP). CMP and CLP further differentiate into multiple myeloid and lymphoid cells, respectively. Lineage decision is an intricate and complex process that, at each stage, is an interplay of cytokines, receptor signaling pathways, and transcription factors.

### SUMOylation and Development and Differentiation of Immune Cells

Emerging trends suggest that SUMO and SUMOylation pathway proteins are essential mediators for developing and activating multiple immune cells, precursors, and the effector population. PIAS1 (protein inhibitor of activated STAT1), a SUMO E3 ligase, also plays a crucial role in maintaining the quiescence of dormant HSCs and balancing the differentiation of HSC between CLP and CMP. Findings from quantitative RT-PCR analysis indicate that Gata1, an essential transcription factor for CMP maintenance, is inappropriately induced in HSCs and CLPs upon *Pias1* disruption, thus inducing the expression of myeloid-specific genes in CLPs and other lineage-negative progenitors simultaneously, repressing B cell-specific genes. Therefore, *Pias1*^–/–^ mice have reduced B cell population, suggesting that PIAS1 is essential for CLP and B-cell progenitors’ survival under homeostatic conditions (Liu et al., 2014). PIAS1 SUMO ligase regulates the self-renewal and differentiation of HSCs. Indeed in B-cell lymphoma, PIAS1 has been shown to assist the SUMOylation of MYC proto-oncogene. Increased MYC SUMOylation resulted in its longer half-life and enhanced oncogenic activity leading to the B-cell lymphomagenesis (Rabellino et al., 2016).

Moreover, in *Pias1^–/–^* mice, the percentage of thymic single-positive CD4^+^ or CD8^+^ T cells were slightly elevated. The frequency of both thymic and splenic CD4^+^ Foxp3^+^ natural T regulatory cells (nTreg) was increased, and the number of thymic CD4^+^ Foxp3^+^ nTreg cells was also significantly increased in *Pias1^–/–^* mice, suggesting that PIAS1 negatively regulates nTreg differentiation. Natural Treg cells are critical in establishing peripheral tolerance, particularly to self-antigens. The physiological significance of increased nTreg cells is underscored by the fact that *Pias1^–/–^* mice are resistant to the development of experimental autoimmune encephalomyelitis (Liu et al., 2010). PIAS1, in addition to facilitating SUMOylation, also regulates transcription by binding to chromatin, thereby repressing transcription. In Foxp3^+^ T regulatory (Treg) cells, PIAS1 maintains a repressive chromatin state of the Foxp3 promoter by recruiting DNA methyltransferases (DNMTs) and heterochromatin protein 1 (HP1) to promote epigenetic modifications (Liu et al., 2010). Alterations in the SUMO ligase activity of PIAS1 bring a change in the SUMOylation levels. This variation can be linked with the transcriptional regulation in HSCs and T cells. System-wide quantitative SUMO proteomics performed with HeLa and HEK293 cells have identified many potential substrates of PIAS1 involved in transcription regulation pathways and cytoskeleton organization (Li et al., 2020). Interestingly, cytoskeleton reorganization is one of the first and crucial events in most immune cell activation. It is quite likely that the immune pathway-specific defects observed in PIAS1-deficient mice are because of the altered SUMOylation level of transcription factors and other substrates, including ones involved in cytoskeleton organization.

Direct evidence that SUMO proteins and pathways play crucial roles in immune cells came from studies utilizing cell type-specific knockout or transgenic mice. For example, overexpression of SUMO2 in T cell-specific transgenic mice resulted in T cell differentiation to interleukin 17 (IL17) producing CD8^+^ T cells, implying that SUMO2-mediated pathways play a critical role in governing the T-cell responses to the pathogens. In these mice, overexpression of SUMO2 increased IL6 production in T cells, IL6-dependent induction of STAT3 phosphorylation, and differentiation of the T cells to IL17-producing CD8^+^ T cells. IL17-producing CD8^+^ T cells are efficient killers of the tumor cells; consequently, these transgenic mice have significantly reduced tumor growth (Won et al., 2015). These findings also indicate that the role of SUMO in regulating tumor growth could partially result from direct action on host immune cells, in particular CD8^+^ T cells. CD4^+^ T helper cells are central players in host immune response that differentiate into specific effector cell types, such as Th1, Th2, Th17, and Treg, depending on the signals generated from cytokines within the immediate milieu. Each of these effector cell types has a distinct and discrete role in governing the immune response. Th1 and Th17 cells are crucial in the efficient clearance of the pathogens, but their aberrant activation can cause severe autoimmunity. T cell lineage-specific deletion of SENP2 in Senp2f/f × Lck-cre mice had more Th1 and Th17 cells in steady state than those in WT mice. Consequently, these mice had higher interferon-gamma levels (IFNγ) and IL17, leading to the development of autoimmune colitis (Yang et al., 2020). Treg cells are another subtype of T helper cells; Treg cells are suppressor T cells required for culminating the immune responses, maintaining peripheral tolerance, and preventing autoimmune disorders.

The specific role of SUMOylation in Treg cells was shown in mice where *ubc9* was selectively deleted in Treg cells. The *ubc9*-deficient Treg cells had defective homeostatic proliferation, impaired activation, and reduced suppressor activity. Deletion of *ubc9* in Tregs led to fatal early onset of autoimmune disorder with increased activated CD4 T cell number, higher secretion of inflammatory cytokines, increased antibodies including anti-dsDNA autoantibodies, and severe infiltration of activated lymphocytes in multiple organs. Tregs with *ubc9* deficiency showed diminished activation; downregulated suppressor molecules such as CTLA4, PD-1, and ICOS; and remarkably reduced production of the suppressor cytokine IL10 (Ding et al., 2016). Severe defects in *ubc9*-deficient Tregs are attributed to the defective TCR signaling leading to a lack of SUMOylation and a consequent decrease in the stability and activity of transcription factor IRF4 (Ding et al., 2016).

Treg cell-specific deletion of *Senp3*, causing a global increase in SUMOylation, enhanced T-cell activation, autoimmune activation, and T cell-mediated antitumor responses. SENP3 is a regulator of Treg cells that functions by controlling the SUMOylation and nuclear localization of a highly conserved repressor, BTB, and CNC homolog 2 (BACH2). BACH2 controls the terminal differentiation and maturation of both B and T lymphocytes. SENP3-mediated BACH2 deSUMOylation prevents the nuclear export of BACH2, thereby repressing the genes associated with CD4^+^ T effector cell differentiation and stabilizing the genes associated with Treg differentiation (Yu et al., 2018).

## SUMOylation in Lymphoid Development

SUMOylation also plays a vital role in lymphoid development. During the early stages of T- and B-cell development, the SUMO protease SENP1 is highly expressed. Following SENP1 deficiency in mice, severe defects in both T- and B-cell development were observed. When compared with the WT littermates, thymi of SENP1^–/–^ had reduced size and cellularity. Moreover, the CD4 and CD8 double-negative (DN) T-cell precursors were also decreased.

Additionally, the B cells’ precursors from various B-cell development stages were also markedly decreased upon SENP1 deficiency. SENP1 depletion in these mice resulted in the accumulation of SUMOylated STAT5, which inhibits STAT5 activity. STAT5 is critical for the development and function of immune cells, and deficient STAT5 activity causes severe defects in both T- and B-cell development. SENP1, therefore, regulates lymphoid development by balancing the level of SUMOylated STAT5 in lymphocytic precursors (Van Nguyen et al., 2012). The fact that SUMOylation plays a vital role in the development of the T cells was further highlighted in mice with selective deletion of *ubc9* in T cells. T cell-specific *ubc9* knockout mice have significantly reduced CD4 and CD8 single-positive T-cell population in both thymus and peripheral lymphoid tissues. In particular, these mice showed defects during the transition from double-positive stage to single-positive cells, highlighting that *ubc9* deficiency results in defective positive selection. Moreover, in *ubc9-*deficient mice, NKT cells and FOXP3^+^ regulatory T cells were also significantly reduced, whereas no difference between γδ T cells was found between WT and *ubc9-*deficient mice (Wang A. et al., 2017). In addition to affecting T-cell development, SUMOylation has also been shown to modulate B-cell development and differentiation.

Interferon-induced protein Daxx plays an important role in IFN-mediated suppression of B-cell development. Daxx gets SUMOylated and translocated to the nucleus of the precursor B cells and suppresses its further progression to mature B cells upon IFN response (Muromoto et al., 2006). Transcriptional repressor B lymphocyte-induced maturation protein-1 (Blimp-1) is the master regulator of plasma cell differentiation. SUMOylation of Blimp-1 is facilitated by SUMO E3 ligase PIAS1 inducing proteasome-mediated degradation of Blimp-1 (Shimshon et al., 2011). Single point mutant of Blimp-1 (K816R mutant), rendering it SUMOylation deficient, poorly interacts with histone deacetylase 2 (HDAC2). Reduced Blimp1–HDAC2 interaction suppresses Blimp-1-mediated transcriptional repression activity and impairs the differentiation of B cells to plasma cells (Ying et al., 2012).

Together, these observations highlight that SUMOylation of several transcription factor proteins and genetic perturbation affecting SUMO dynamics play highly cell type-specific and pleiotropic roles in host immune cell development.

## SUMOylation in Immune Cell Activation

SUMOylation is one of the dynamic and reversible signaling events that cause changes in the three-dimensional structure of the target protein, creating a scaffold for the downstream signaling cascade. Immune cells face the enormous challenge of recognizing, responding, and remembering the ever-evolving plethora of pathogens. Importantly, immune cells can cater to their responses as per the pathogen. These individualized catered responses are attributed to the immune cell’s ability to recruit discrete signaling proteins and initiate distinct signaling pathways. Phosphorylation–dephosphorylation cascade is one of the major signaling known in immune cell signaling, and recent studies indicate the role of SUMOylation in immune cell signaling. Multiple different receptors have been shown to induce SUMOylation of one or more targets in many immune cells. These targets include transcription factors, kinases, adaptor proteins, and specific receptors.

One of the critical transcription factors in immune cells is the nuclear factor of activated T cells (NFAT), which is highly expressed in most immune cells. In T cells, NFAT regulates the differentiation of various effector cell subsets by governing the expression of their lineage-specific transcription factors. Additionally, NFAT also regulates the transcription of their signature cytokines and their receptors. Given the central role of NFAT in modulating the antigen-mediated T cell responses, it is one of the tightly regulated proteins. Many isoforms of NFAT are expressed in T cells. The constitutively expressed isoform, NFATc1/C, is highly SUMOylated. The SUMOylated NFATc1 is translocated to promyelocytic leukemia (PML) bodies in the nucleus, leading to the deacetylation of histones and suppression of the interleukin-2 gene *in vitro* (Nayak et al., 2009). Recently, a role for *in vivo* NFATc1 SUMOylation is reported in the transgenic mouse in which SUMO modification of NFATc1 was blocked.

Interestingly, these mice had significantly high IL2 production and enhanced Treg cell proliferation, and suppressed IL17 and IFNγ release. Consequently, these mice were protected from experimental autoimmune encephalomyelitis and graft-versus-host disease (Xiao et al., 2021). TCR signaling induces the phosphorylation and activation of another transcription factor, JunB, which then translocates to the nucleus and results in the expression of various T cell-associated cytokine genes like IL-2, IL-4, and IL-10. JunB is a target for SUMOylation in many cell types, including T cells. Blocking JunB SUMOylation reduced the transactivation of IL-2 and IL-4 in T cells (Garaude et al., 2008).

c-Maf is another transcription factor whose function is dependent on SUMOylation. c-Maf SUMOylation in CD4^+^ T cells has been shown to regulate IL-21-mediated diabetes in NOD mice in an inverse manner. T cell-specific transgenic NOD mice overexpressing SUMOylation site-mutated c-Maf developed diabetes more rapidly than Tg-WTc mice in a CD4^+^ T cell-intrinsic manner. SUMO-defective c-Maf transactivated *Il21*, resulting in increased levels of IL21 in T cells, resulting, in turn, in the induction of diabetes. This increase in IL-21 levels was associated with an increased concentration of SUMOylated c-Maf (Hsu et al., 2018).

TCR stimulation alters various SUMO pathway proteins; for example, TCR and CD28 stimulation in Treg cells leads to SENP3 accumulation. Following TCR stimulation, Phospholipase C-γ1 (PLC-γ1) gets SUMOylated and forms microclusters containing TCR signalosome, which mediates T-cell activation. DeSUMOylation of PLC-γ1 prevents microcluster formation and, therefore, blocks T-cell activation (Wang et al., 2019). T-cell activation is dependent on a central adaptor protein, SH2 domain-containing leukocyte phosphoprotein of 76 kDa (SLP-76). T-cell stimulation results in Ubc9-mediated SLP-76 SUMOylation. Moreover, TCR signaling-mediated SLP-76 SUMOylation is crucial for the SUMOylation of NFAT, resulting in IL-2 transcription (Xiong et al., 2019).

T-cell activation is initiated upon recognizing the antigen–MHC II complex presented on the antigen-presenting cells (APCs). Interaction between the T cells and APC generates the immunological synapse. The strength, quality, and composition of the immunological synapse define multiple parameters of the T-cell activation. Protein kinase C-θ (PKC-θ) is localized in the immunological synapse following TCR signaling, where it also interacts with T-cell co-stimulatory molecule CD28 and filamin A resulting in amplification of TCR signaling. Interestingly, the localization of PKC-θ at the immunological synapse following TCR signaling is dependent on its SUMOylation by PIASxβ. DeSUMOylation of PKC-θ blocked its localization to the immune synapse, inhibiting its interaction with CD28 and filamin-A, resulting in dysregulated activation and proliferation of T cells. Moreover, the reduced availability of PKC-θ in immune synapses led to the production of Treg cells (Wang et al., 2015). These results suggest that SUMOylation of various signaling mediators may fine-tune T-cell responses toward creating a balance between pro-inflammatory or anti-inflammatory responses.

The effects of SUMOylation are not limited to the T cells only but are observed in many other immune cell types. For example, in dendritic cells, SUMO-2 overexpression causes downregulation of IL12 by blocking the translocation of the p65 subunit of NF-κB into the nucleus. The translocation of NF-κB is also SUMOylation dependent (Huang et al., 2005; Liu et al., 2012). Decreased IL12 levels modulate signals to the naïve CD4^+^ T cells for inducing Th2-type response (Kim et al., 2011). Table 1 summarizes the effect of SUMOylation on various immune cells.

**TABLE 1:** Mechanism and effects of immune cell protein SUMOylation.

Cell Type	Effect of SUMOylation and the mechanistic details
T cells	DeSUMOylation of STAT5 controls defects in early T cells (Van Nguyen et al., 2012)
	SUMOylation promotes the transition from double-positive to single-positive T cells (Wang A. et al., 2017)
	NFATc1 SUMOylation contributes to subtype-specific lymphokine production and Teff cell proliferation (Xiao et al., 2021)
	JunB SUMOylation leads to IL-2 and IL-4 production (Garaude et al., 2008)
	DeSUMOylation of SMAD4 controls proliferation of Th1 and Th17 cells (Yang et al., 2020)
	SUMOylated c-Maf has epigenetic effects on the increased levels of IL-21 and contributes to Type I diabetes (Hsu et al., 2018)
	SUMOylation of PLC-γ1 mediates TCR activation (Wang et al., 2019)
	SLP-76 SUMOylation is required for IL-2 transcription by SUMOylated NFAT (Xiong et al., 2019)
	SUMOylated IRF4 regulates TCR dependent gene expression (Ding et al., 2016)
	The presence of SUMOylated PKC-θ at immunological synapse maintains a balance between Teff and Treg cells (Wang et al., 2015)
	SENP3 desumoylates BACH2 to stimulate Treg cell-specific genes (Yu et al., 2018)
	PIAS1 suppresses the Treg cell differentiation (Liu et al., 2010)
B cells	DeSUMOylation of STAT5 controls defects in early B cell (Van Nguyen et al., 2012)
	SUMOylated Blimp-1 regulates B cell differentiation into plasma cell (Ying et al., 2012)
	SUMOylated Daxx suppress B cell development (Muromoto et al., 2006)
Dendritic cells	SUMOylated TRIM5α is sequestered in the nucleus in dendritic cells and helps them in immune sensing (Portilho et al., 2016)
	SUMO-2 overexpression induces dendritic cells to shift the CD4^+^ T cells to Th2 type (Kim et al., 2011)

*An extensive list of immune signaling proteins from T cells, B cells, and dendritic cells. These proteins are SUMOylated and are involved in a myriad of functioning of immune cells.*

TLRs on the innate immune cells recognize molecular patterns present on the wide range of pathogens, bacteria, viruses, and fungi. Signaling through TLRs initiates inflammatory responses and also primes the adaptive immune cells. A wide range of cytokines is produced following TLR activation in a context-dependent manner. TLRs are therefore highly regulated at multiple levels, including SUMOylation. Interestingly, the TLR-mediated inflammatory responses are curbed by SENP6 activity. Depletion of SENP6 resulted in the NF-κB-mediated induction of the proinflammatory genes following activation of TLR3, TLR4, and TLR7. TLR-mediated expression of proinflammatory genes is dependent on the NF-κB activation. NF-κB activation requires I-κB kinase, which comprises two catalytic IKKα and IKKβ subunits and a regulatory protein NF-κB essential modifier (NEMO/IKKγ) (Zandi et al., 1997). Interestingly, ubiquitination of NEMO is crucial for NF-κB activation (Tang et al., 2003). Following TLR signaling, NF-κB essential modifier (NEMO/IKKγ) undergoes SUMO-2/3 modification, which prevents NEMO binding to the deubiquitinase CYLD and thus indirectly enhancing the IKK activation. NEMO is deSUMOylated by SENP6, and siRNA-mediated reduction of SENP6 levels in mice resulted in enhanced activation of NF-κB signaling-dependent proinflammatory cytokine production (Liu et al., 2013). NEMO is a target for both SUMOylation and ubiquitination, and how NEMO SUMOylation affects the binding of NEMO with deubiquitinase CYLD and thus the activation of the NF-κB pathway is relatively unclear.

TLR-dependent NF-κB activation is mediated by the TAK1 signalosome consisting of the TRAF6/TAB2/TAK1 complex. The SUMOylation of the TAK1 signalosome component protein TAB2 is enhanced by the SUMO E3 ligase TRIM60 (tripartite motif-containing protein) E3 SUMO ligase (Gu et al., 2020). Interestingly, TAB2 ubiquitination levels are unaffected by TRIM60 overexpression. Observations from the TRIM60 knockout MEFs indicated an enhancement in the MAP kinase signaling pathway and NF-κB activation (Gu et al., 2020).

SUMOylation is known to repress NF-κB-mediated inflammation (Portilho et al., 2016; Hu et al., 2017). In the absence of SUMOylation, engagement of Toll-like receptor 4 with lipopolysaccharide (LPS) increased secretion of NF-κB-dependent inflammatory cytokines and enhanced type I interferon release. However, when SUMOylation is abolished, LPS induces higher and constitutive IFN-β production (Decque et al., 2016). One of the mechanisms by which SUMOylation prevents inflammation is the silencing of Ifnb1 expression. Lack of Ifnb1 inactivates the TLR-induced production of inflammatory cytokines, hence protecting tissue from damage due to prolonged cytokine expression (Qiao et al., 2013).

## Modulation of Host SUMO Pathway by Pathogens

Considering the fact that several of the host factors are modified to mediate immune response, a marked change can be noticed in the global SUMOylation during bacterial and viral infections. Some pathogens develop countering strategies to negate SUMO-mediated host defense as an adaptation. The pathogens either directly target enzymes of the SUMOylation pathway or perturb the SUMOylation dynamics of proteins involved in mounting the immune response against pathogens. Alternatively, some pathogens can utilize the host SUMOylation machinery to modify their proteins, aiding in the amplification and sustenance of the infection.

### Targeting SUMO Pathway Enzymes

**FIGURE 2:**
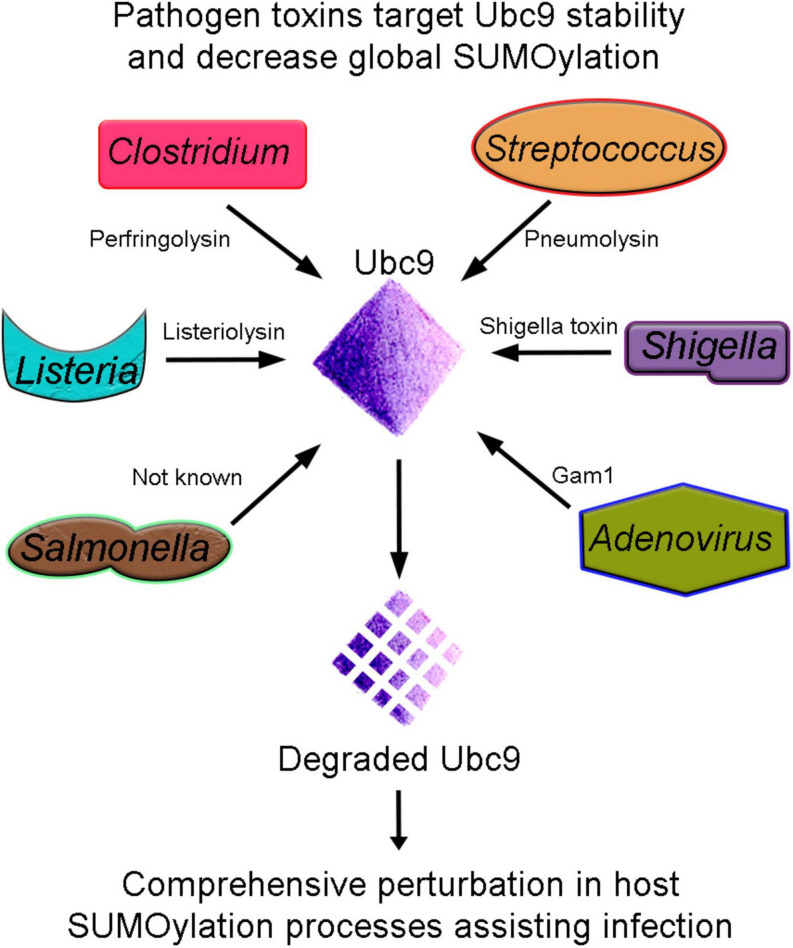
Ubc9 degradation, an effective strategy for pathogenesis. Bacterial (*Listeria*, *Clostridium*, *Streptococcus*, *Shigella*, and *Salmonella*) and Adenovirus pathogens produce toxins that target the host conjugating enzyme Ubc9. Unstable Ubc9 adversely affects the SUMOylation dynamics, and consequently, several vital cellular processes are compromised and assist in infection.

One of the earliest reports about pathogens targeting the host SUMOylation pathway came from *Listeria monocytogenes*. Listeria inflicts listeriosis, a severe food-borne disease in humans. *L. monocytogenes* produces a pore-forming toxin Listeriolysin (LLO). LLOis involved in evading the pathogen’s internalization into host vacuole during infection. Interestingly, it was uncovered that Listeria infection induces a significant decrease in overall SUMOylation of host proteins. The detailed analysis further indicated that the LLO toxin catalyzes the degradation of Ubc9, the conjugating enzyme of the pathway. The strategy of targeting Ubc9 stability is efficient and effective as many other pathogens like *Clostridium perfringens* and *Streptococcus pneumoniae* secrete perfringolysin (PFO) and pneumolysin (PLY), respectively, and compromise the stability of Ubc9. Degraded Ubc9 imparts a decrease in overall host SUMOylation (Ribet et al., 2010). Ubc9 is also depleted in *Shigella* spp.*-*infected cells (Sidik et al., 2015). Ubc9 is the unique and indispensable enzyme of the SUMOylation pathway; thus, targeting Ubc9 seems an obvious choice for pathogens. *Shigella* spp. cause shigellosis and inflict a diarrheal disease by invading the colon epithelium. The infection activates a robust inflammatory response damaging the gut tissue. SUMOylation was shown to activate an innate immune response against *Shigella* invasion by mediating the induction of a defined transcriptional paradigm (Fritah et al., 2014). The type 3 secretion system (T3SS)-mediated delivery of *Shigella* toxin and subsequent infection also induces proteasome-mediated Ubc9 degradation as a successful infection strategy leading to decreased global SUMOylation in infected cells (Figure 2 and Table 2).

**TABLE 2:** List of parasites modulating host SUMOylation machinery and host or parasite effector protein SUMOylation.

Pathogen	Mechanism of modulation of the host SUMO pathway
*Listeria monocytogenes*	Listeriolysin toxin degrades Ubc9, causing a global decrease in the host SUMOylation (Ribet et al., 2010)
*Clostridium perfringens*	Degradation of Ubc9 by Perfringolysin toxin (Ribet et al., 2010)
*Streptococcus pneumoniae*	Degradation of Ubc9 Pneumolysin toxin (Ribet et al., 2010)
*Shigella* spp.	Causes T3SS mediated proteasomal degradation of Ubc9 (Sidik et al., 2015)
Adenovirus	Degrades Ubc9 and inhibits SAE1/SAE2 (Boggio et al., 2004)
*Salmonella typhimurium*	Downregulates Ubc9 via miR30 (Verma et al., 2015)
Kaposi Sarcoma herpes virus	Represses expression of SENP6 via Latency-associated nuclear antigen (LANA) (Lin et al., 2017)
Hepatitis B virus	Hbx protein causes deSUMOylation of SP110 of PML NBs (Sengupta et al., 2017)
Herpes Simplex virus	ICP0 protein causes proteasomal degradation of SUMOylated proteins like PML via SIM interaction (Boutell et al., 2011)
Epstein Barr Virus	BLZF1 protein depletes SUMOylated PML by competing for SUMO1 and limiting its abundance (Mauser et al., 2002)
Cytomegalovirus	IE1 protein abrogates SUMOylation of Sp100 and PML (Müller and Dejean, 1999)
Human Papillomavirus	SUMOylation of viral E2 protein regulates its transcriptional function and inhibits its ubiquitination and degradation (Wu et al., 2009)
SARS-COV	SUMOylation of viral N protein aids in its homo-oligomerization (Li et al., 2006)
Ebola Zaire Virus	VP35 triggers SUMOylation of IRF3 and IRF7, leading to downregulation of interferon signaling pathways (Kubota et al., 2008; Chang et al., 2009). SUMOylation of VP24 prevents its degradation (Vidal et al., 2020).
Avian Influenza virus H5N1	SUMOylation of viral protein NS1 prevents its degradation (Xu et al., 2011)
*Anaplasma phagocytophilum*	SUMOylation of AmpA helps in pathogen survival (Beyer et al., 2015)
*Ehrlichia chaffeensis*	SUMOylation of TRP120 aids in its recruitment and interaction with the host (Dunphy et al., 2014)

*The table provides an account of pathogens, toxin molecules produced, and the perturbation in SUMOylation dynamics induced either by Ubc9 instability or by targeting other enzymes/components of the SUMO pathway.*

Furthermore, *Salmonella typhimurium*, causing typhoid fever/self-limiting gastroenteritis, presents another example of a pathogen affecting Ubc9 stability as an infection strategy. Interestingly, the Ubc9 degradation seems to be an indirect consequence of pathogen toxin. Instead, it results from the activation of the miR30 family of miRNA (Verma et al., 2015). Moreover, *S. typhimurium* compounded problems with the SUMOylation process and host defense by affecting the E3 ligase enzyme PIAS1. Thus, an overall reduction in SUMOylation due to synergistic loss in Ubc9 and PIAS1 function seems to be another infection strategy (Table 2).

Early reports from viruses indicated SUMOylation pathway targeting as an additional node. The adenoviral protein, Gam1, activates transcription in host cells to favor the successful infection. The overall host SUMOylation levels and Gam1-dependent transcriptional activation are inversely related. Gam1 inhibits host SUMOylation by proteasomal degradations of Ubc9 and inhibition of SAE1/SAE2 enzyme’s catalytic activity. Thus, Gam1 exploits the SUMO pathway to activate the transcription of genes assisting adenoviral replication (Boggio et al., 2004). Thus, several pathogens target the Ubc9 stability, affect the overall cellular SUMOylation, and derail host immune pathways to establish infection (Figure 2 and Table 2).

Shifting SUMOylation equilibrium in either direction is sufficient to induce perturbance in cellular homeostasis. SENPs are the class of SUMO proteases required for maintaining the dynamic cycle of SUMOylation (Nayak and Müller, 2014). Thus, modulating the function of SENP has a direct consequence on SUMOylation of key host immunity factors. Pathogens are working through SUMOylation level modulation and can also target SENP enzymes to prepare the host environment favorable for pathogenesis. In this context, it was reported that the Kaposi Sarcoma Herpesvirus (KSHV) infects B cells and can often induce malignancies in AIDS patients. One of the major proteins in KSHV, Latency-associated nuclear antigen (LANA), is required to maintain the latent phase of the virus. LANA binds SENP6 promoter and represses its expression, an effect capable of increasing global SUMOylation and polymeric chain formation on SUMO targets. LANA itself is a SUMO target, and in its SUMOylated state, it cannot establish latency. Thus, LANA regulates its own abundance in the latent phase of the virus pathogenesis by repressing SENP6 (Lin et al., 2017). Several reports bring forth the idea that SUMOylation can serve as a primer for ubiquitination, thus controlling the turnover of proteins. In this context, it becomes crucial to highlight the fact that the interplay of SUMOylation and ubiquitination is a critical determinant of immune signaling and immune activation events.

### Pathogens Affecting PML Nuclear Bodies

PML antigens and proteins like SP100, SP110, and ND10 form the PML nuclear bodies (PML NBs). They are located in the nuclear matrix and regulate several nuclear functions such as replication, transcription, and epigenetic gene silencing. PML protein SUMOylation is known to be critical for PML nuclear body assembly, stability, and function. Cellular stress, viral infections, DNA damage, and oxidative stress modulate the regulation of PML NBs. PML protein SUMOylation is known to be critical for PML nuclear body assembly, stability, and function. PML NBs are upregulated during several viral infections and are thought to have antiviral properties (Lallemand-Breitenbach and de Thé, 2010). During pathogenesis and disease conditions, like acute PML, PML-RARα protein is SUMOylated and degraded and thus affects the integrity of PML NBs. Hence, some pathogens have developed strategies to dysregulate the PML bodies by effectively hindering their SUMOylation (Everett and Chelbi-Alix, 2007).

SP110 is a chromatin and transcriptional regulator that, in close association with PML proteins, resides in the PML NBs. The SUMO1 modification of SP110 is necessary for its residence in the PML NBs. SP110, an important player of B-cell immortalization, interacts with Epstein–Barr virus (EBV) protein EBNA-LP. This impacts the stability of PML NBs and the regulation of gene expression (Sengupta et al., 2017). Another EBV protein, BLZF1, is SUMOylated and induces PML NB dispersion. However, the SUMOylation-deficient BLZF1 protein only partially affects the stability of PML bodies (Hagemeier et al., 2010).

The intrinsic SUMO pathway of the host generates an antiviral response against Herpes Simplex Virus 1 (HSV1). Infected cell protein 0 (ICP0) from HSV1 has the E3 ubiquitin ligase activity. Functionally, ICP0 is a SUMO targeting Ubiquitin ligase (STUbL), which identifies SUMO-modified proteins *via* SIMs and, through the E3 ubiquitin ligase activity, ubiquitinates target proteins and sends them for proteasomal degradation. ICP0 degrades SUMOylated PML in a SIM-dependent manner and thus affects the stability of PML NBs. Host cells infected with ICP0-depleted HSV-1 elicited an antiviral response *via* the SUMO pathway, which played a key role in perturbing the infection of the virus (Boutell et al., 2011). The Immediate early 1 (IE1) protein of Cytomegalovirus (CMV) is a target for SUMO1 modification. Importantly, IE1 induces deSUMOylation of PML and Sp100. DeSUMOylated PML and Sp100 induced disruption of PML-NBs, thus paving the way for viral pathogenesis (Müller and Dejean, 1999).

### Pathogens Using SUMO Modification for Pathogenesis

Pathogens have resorted to modulate SUMOylation of a particular protein or overall cellular host SUMOylation as a successful infection strategy. They target enzymes mediating SUMO conjugation or deconjugation processes. Some pathogens also resort to modifying or preventing their proteins from getting SUMOylated as an infection strategy.

Human Papillomavirus (HPV) protein E2 is required for host genome regulation and viral replication. The HPV16 E2 protein is efficiently SUMOylated by host SUMO machinery. SUMO modification at lysine 292 (K292) residue in the DNA binding domain is required for regulatory functions of E2. The K292R mutation in the E2 protein rendering it SUMOylation deficient negatively affected the transcriptional activity (Wu et al., 2008). More importantly, upregulation of the SUMO pathway stabilized HPV16 E2 protein levels, and SUMOylation prevented proteasome-mediated degradation of E2. This observation hints at the importance of SUMOylation in the functioning of regulatory viral proteins in effecting pathogenesis (Wu et al., 2009).

The SARS-CoV nucleocapsid N-protein has RNA binding domains and regions required for self-assembly and homo-oligomerization. N-protein was found to interact with Ubc9 and efficiently SUMO modified. Interestingly, the SUMOylation of the N-protein at lysine residue (K62) in the RNA binding domain helped in its homo-oligomerization, an event critical for nucleocapsid assembly and SARS-CoV infectivity (Li et al., 2006). Ebola Zaire virus inflicts highly pathogenic outbreaks in humans by efficiently replicating inside macrophages and dendritic cells and suppresses the interferon signaling in these cells. VP35 protein of Ebola virus interacted with Ubc9 and PIAS1 proteins and induced IRF7 SUMOylation and interferon expression. VP35 did not affect NF-κB activity and, thus, proinflammatory cytokine expression. Through these interactions, VP35 used host SUMOylation machinery to its benefit by blocking interferon responses (Kubota et al., 2008; Chang et al., 2009). VP24, a minor matrix protein from the Ebola virus, interacted non-covalently with SUMO and was covalently modified. SUMOylation-deficient VP24 protein had a reduced effect on blocking the interferon pathway. Thus, viral proteins do exploit the SUMOylation pathway to establish successful infections (Vidal et al., 2020). Non-structural protein 1 (NS1) of avian influenza virus H5N1 is required for a high virulence rate. The NS1 protein interacted with Ubc9 and was efficiently modified at the carboxy-terminal end by SUMO1. SUMOylation-deficient NS1 is unstable and susceptible to degradation. This observation further established that critical viral proteins can exploit host SUMOylation machinery interactions and SUMOylation for their benefit in establishing the infection (Xu et al., 2011).

In this growing list of organisms and pathogens utilizing host SUMOylation machinery to their benefit when establishing the infection and battling the hostile cellular milieu of the host is *Anaplasma phagocytophilum*. An obligate intracellular parasite, *A*. *phagocytophilum* infects polymorphonuclear cells like Neutrophil. The effector protein of Anaplasma, AmpA, is critically required for the pathogen’s survival inside the host. Vacuolar membrane-localized AmpA is SUMOylated, and the same is critically required for pathogen infection. Inhibition of SUMOylation (pharmacological) by anacardic acid significantly reduced the bacterial load in cells. *Anaplasma*, harboring SUMOylation-deficient AmpA, was less potent in its infectivity. This study suggested that SUMOylation of effector proteins is a way for pathogens to establish pathogenesis and survive (Beyer et al., 2015). Similarly, *Ehrlichia chaffeensis*, belonging to the same family as *A. phagocytophilum*, also utilizes the host SUMO pathway to modify the Type I secretion system effector protein, TRP120. Inhibition of SUMOylation affected TRP120 interaction and *Ehrlichia* replication. This observation presents another example of SUMOylation of pathogen effector proteins by host machinery as a strategy for infection and pathogen survival (Dunphy et al., 2014).

## SUMO Regulation of Host Proteins Involved in Immunity

Various host proteins are SUMOylated for efficient defense against pathogens. One of them is TRIM5α, a restriction factor that blocks the incoming retrovirus infections. SUMOylation of TRIM5α leads to its sequestration in the nucleus of dendritic cells and rendering it inactive. Microscopy results show that TRIM5α colocalizes with the Cajal bodies in the dendritic cell nucleus. TRIM5α cannot efficiently recognize and restrict the retroviral RNA that is present in the cytoplasm. This helps in sensing viral components by host sensors, which trigger the type-I IFN pathway, crucial for viral clearance. If TRIM5α is not SUMOylated, it will restrict the viral DNA replication, and sensor proteins will not be able to trigger the IFN production for controlling the viral spread (Portilho et al., 2016). TRIM5α restricts the entry of N-tropic murine leukemia virus (N-MLV) in HEK293T cells by interaction with SUMOylated retroviral capsid (CA) through its two SIMs. Mutations in CA decrease the interaction between SIMs and hence the antiviral activity. CA must contain Ubc9 interaction sites and SUMOylatable lysine residues for interaction with TRIM5α, thus restricting the virus (Arriagada et al., 2011).

Vesicular stomatitis virus infection causes SUMOylation of transcription factors IRF3 and IRF7. IRF3 and IRF7 also get SUMOylated following TLR and RIG-I/MDA-5 signaling pathways (Hu et al., 2017). SUMOylation of IRF3 and IRF7 acts as a negative regulator of the type-I interferon pathway leading to reduced interferon production. SUMO-mediated downregulation of interferon and inflammatory cytokine expression is crucial for preventing host cytotoxicity and tissue injury. Host Tripartite motif-containing protein 38 (TRIM38) SUMOylates RIG-I and MDA5 upon RNA virus infection. RIG-I and MDA5 triggers the expression of downstream antiviral genes like *IFNB1*, *CXCL10*, and *TNFA.* However, at later stages of infection, SENP2 mediates deSUMOylation to stop interferon production and prevent unnecessary damage to the host cells (Kubota et al., 2008).

The human Myxovirus resistance protein A (MxA) belongs to the family of large GTPase proteins. Its expression in response to interferon pathway induction by viral infection is crucial for protection against many viruses, including vesicular stomatitis virus (VSV). The SUMOylation of MxA increases its stability and oligomerization capacity and inhibits the VSV gene transcription but has no effect on the virus entry into the cell. Knockdown of MxA in SUMO-expressing cells abrogated the VSV resistance, implying that MxA is an important player in SUMO-mediated resistance to VSV (Maarifi et al., 2016).

Gag protein of Human Immunodeficiency Virus type-1 (HIV-1) interacts with the host SUMO-1 and E2-conjugating enzyme, Ubc9, through the p6 domain. The p6 domain facilitates the virion assembly and budding from the infected cells. SUMOylation at the N-terminal K27 residue is critical as the K27R mutation rendering p6 non-SUMOylatable leads to an increase in infectivity of the released virions. Overexpression of SUMO-1 along with Ubc9 and SUMO-1 alone reduces the infectivity of viruses. The reason for the reduced infectivity of virions is not known (Gurer et al., 2005).

The switch from latent to lytic viral transcription is mediated by the EBV immediate-early protein BZLF1. BZLF1 plays a role in lytic gene transcription and genome replication. During EBV infection, BZLF1 is SUMOylated at the lysine amino acid residue at 12 by SUMO-1, 2, or 3. BZLF1 serves as a transcriptional activator, but it is inhibited due to SUMOylation; hence it results in viral latency due to low expression of lytic genes. EBV-encoded protein kinase (EBV-PK) inhibits BZLF1 protein SUMOylation during viral reactivation from latency (Hagemeier et al., 2010).

## SUMO in Interferon Signaling

IFNs are important cytokines released by immune cells upon pathogenic insult. Many different immune receptors, specifically PRRs, upon binding to their ligands induce IFN production. Although IFNs were originally recognized for their antiviral properties, they also have multiple immunomodulatory functions. Therefore, IFN responses are tightly regulated by multiple molecular mechanisms, including SUMOylation. Interestingly, signaling pathways induce the production of IFN and downstream signaling proteins. These proteins induced upon IFN signaling are regulated by SUMOylation machinery, emphasizing the role of SUMO in IFN responses.

Three types of IFNs are reported in humans: type I IFN, which includes IFNα, IFNβ, IFNε, IFNκ, and IFNω; type II IFN where the only member is IFNγ; and type III IFN, which consists of IFNλ1 to IFNλ4. IFNs are recognized by their respective receptors, which initiate the JAK/STAT pathways, ultimately resulting in the transcription of IFN stimulated genes (ISGs). SUMOylation of STAT1 acts as a negative regulator as in the absence of SUMOylation, STAT1 has prolonged DNA-binding activity and nuclear localization in response to IFNα stimulation (Ungureanu et al., 2005). SUMOylation of IFN response is required to avoid unnecessary activation of cells against endogenous and self-nucleic acids. Indeed, the loss of SUMOylation resulted in a potent type-I IFN response even in the absence of external stimuli through a hitherto unknown non-canonical mechanism. Constitutive type-I IFN response, therefore, led to autoimmune disorders. This spontaneous IFN response was negatively regulated by SUMO2 and SUMO3 (Crowl and Stetson, 2018).

TLR signaling and IFNα treatment induce global cellular SUMOylation and increased expression of major SUMO paralogs. Accordingly, the expression of SUMO paralogs was controlled by RNA binding protein, Lin28, and microRNA Let7. The IFN/Lin28/Let7 axis enhanced overall cellular SUMOylation levels, and IFN-triggered hyperSUMOylation blocked the replication of two retroviruses, HIV and murine leukemia virus (B-MLV). However, inhibition of SUMOylation and IFN treatment could not curb virus replication, highlighting that IFN-mediated global cellular SUMOylation contributes against viral pathogenesis. This global increase in protein SUMOylation is also dependent on the PML protein (Sahin et al., 2014). PML, also known as TRIM19, owing to its SUMO E3 ligase activity, mediates the SUMOylation of many PML NB-associated proteins. IFN signaling induces PML-dependent Ubc9 translocation to the nuclear matrix, leading to its recruitment to PML NBs. The increase of PML expression and the recruitment of Ubc9 within PML NBs promote the enhancement of SUMOylation in response to IFN, creating an amplification loop (Arriagada et al., 2011).

**FIGURE 3:**
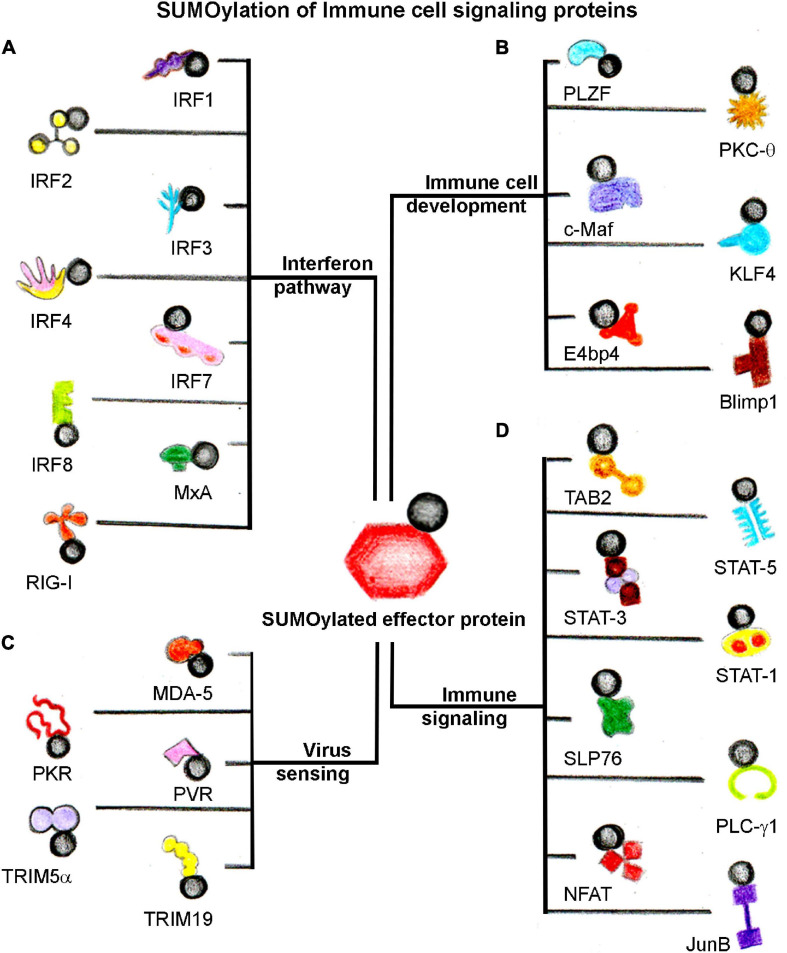
SUMOylation of host immune signaling proteins. SUMOylation dynamics of several host immune cell proteins alter in response to infection. A general categorization of SUMOylated target host proteins involved directly in **(A)** Interferon signaling, **(B)** Immune cell development, **(C)** Virus sensing, and **(D)** Immune signaling events. The black circle represents SUMO.

Through these arguments, it can be suggested that SUMOylation dynamics controlled by conjugation and deconjugation processes play a critical role in sensing viruses and mounting an appropriate interferon response. An extensive array of host immune signaling, immune cell development, viral sensing, interferon-pathway proteins, and specific pathogenic proteins are SUMOylated to orchestrate the efficient host immune responses during host–pathogen interaction (Figure 3 and Table 3).

**TABLE 3:** Immune cell target proteins, SUMOylation, and functional significance.

Immune Cell Target	Consequences of immune cell target protein SUMOylation
Blimp-1	Regulate its intracellular stability and B cell differentiation (Shimshon et al., 2011; Ying et al., 2012).
E4bp4	Regulates NK cell development (Kostrzewski et al., 2018).
IRF-1	Its SUMOylation attenuates the transcriptional activity (Park et al., 2007).
IRF-2	Regulate its transcriptional activity in two ways- increases its ability to inhibit IRF-1 transcriptional activity, decreases its ability to activates the ISRE and H4 (Han et al., 2008)
IRF-3	Part of host immune response against the pathogen and negatively regulates IFN transcription (Kubota et al., 2008)
IRF-4	Regulate its intracellular stability and functions in Treg cells (Ding et al., 2016)
IRF-7	Part of host immune response against the pathogen and negatively regulates IFN transcription (Kubota et al., 2008)
IRF-8	Regulate innate immune response (Chang et al., 2012)
Jun B	Transactivation of IL-2 and IL-4 in T cells (Garaude et al., 2008)
KLF4	Regulate IL-4 induced macrophage M2 polarization by increasing its transcriptional activity (Wang et al., 2017)
MDA-5	SUMOylation MDA-5 regulates its stability in uninfected and early RNA virus-infected cells (Hu et al., 2017)
MxA	Two SIM’s in MxA are essential for its antiviral activities; SUMOylation is not essential for antiviral activities (Brantis-de-Carvalho et al., 2015)
Myc	Regulate its stability and half-life, B cell lymphomagenesis (Rabellino et al., 2016)
NFAT	Regulate IL-2 Transcription (Nayak et al., 2009; Xiao et al., 2021)
PKCθ	Regulate T cell proliferation (Wang et al., 2015)
PKR	Regulate its activation and stability upon viral infection (Shimshon et al., 2017)
PLC-γ1	Controls PLC-γ1-mediated T cell activation (Wang et al., 2019)
PLZF	Represses the transcriptional activity of the IL-3 receptor alpha chain (Kang et al., 2003)
PVR	Regulating the recognition and killing of tumor cells by NK cells (Zitti et al., 2017)
RIG-I	Regulating its stability in uninfected and early infected cells (Mi et al., 2010; Doiron et al., 2017; Hu et al., 2017)
SLP76	Regulating IL-2 transcription (Xiong et al., 2019)
STAT-1	Attenuating cell sensitivity to IFN-γ by Inhibiting STAT1 phosphorylation, it’s binding to DNA, and the transcription of specific ISGs (Rogers et al., 2003; Maarifi et al., 2015)
STAT-3	Negatively regulates its activity by promoting its interaction with TC45 in the nucleus (Zhou et al., 2016)
STAT-5	Regulate the development and function of immune cells (Van Nguyen et al., 2012)
TAB2	Negatively regulates its function as an adaptor for JNK, inhibition of MAPK and NF-κB pathways (Wang et al., 2014; Gu et al., 2020)
TRIM5α	SIM’s are required for its antiviral activity, and SUMOylation is for localization (Arriagada et al., 2011; Dutrieux et al., 2015; Portilho et al., 2016)
TRIM19	Nuclear localization and antiviral responses (El Asmi et al., 2014)

*A detailed account of immune cell target protein SUMOylation and functional consequences on immune signaling, cell development and differentiation, virus sensing, and antiviral pathway activation.*

## Conclusion

Ever-evolving pathogens pose a continuous challenge to the host. Therefore, the host immune system employs a wide range of cellular and molecular players, which work in synergy to combat the pathogen. In the network of host players that govern immune responses, SUMO machinery has found a profound role. The importance of the SUMO proteins and pathways in the regulation of the host immune response is underscored by the fact that they are indispensable for most aspects of immunity, including maintenance and differentiation of HSCs, development and activation of immune cells, the establishment of pathogenesis, and protection from pathogens. Lessons learned from the knockout and transgenic studies indicate the essential and multifaceted role of SUMOylation in autoimmune and autoinflammatory disorders. However, the mechanisms underlying these observations remain poorly understood. Furthermore, studies directed at immune cells exploring SUMOylation in greater detail are needed to better understand distinct proteins and specific pathways whose components are SUMO modified in discrete immune cell types in a context-dependent manner. Knowledge obtained from these studies will pave the way for designing novel therapeutics by targeting SUMO machinery and governing immune modulation. Considering the importance of SUMO modification of several host factors with prominent roles in mounting, propagating, and modulating the entire immune response to pathogen infection, studies identifying the SUMOylome of the immune system are around the corner and will benefit researchers.

## Author Contributions

STK, GJ, PA, VM, AC, and RKM developed the idea, collected information, and wrote and edited the manuscript. AC and RKM supervised manuscript development and arranged the funding. All authors contributed to the article and approved the submitted version.

## Conflict of Interest

The authors declare that the research was conducted in the absence of any commercial or financial relationships that could be construed as a potential conflict of interest.
